# Comparison of Missing School Meals among Public Schools: How Did New York State Do during COVID-19?

**DOI:** 10.3390/ijerph19105838

**Published:** 2022-05-11

**Authors:** Amanda A. Harb, Katherine J. Roberts, Julia E. McCarthy, Pamela A. Koch

**Affiliations:** 1 Department of Health and Behavior Studies, Teachers College, Columbia University, New York, NY 10027, USA; aah2207@tc.columbia.edu (A.A.H.); kjr20@tc.columbia.edu (K.J.R.); 2 New York State Health Foundation, New York, NY 10018, USA; mccarthy@nyshealth.org

**Keywords:** school meals, school lunch, school breakfast, summer meals, participation, COVID-19

## Abstract

Background: The COVID-19 pandemic created barriers to participation in school meals. As a result, many students may have missed out on school meals. The objectives of this study are (1) to compare the number of school meals served by New York State public schools during the first spring and summer of the COVID-19 pandemic to the number served before the COVID-19 pandemic, and (2) to determine relationships between the number of meals served and the levels of school district need and urbanicity. Methods: This study is a secondary analysis of administrative data. The percentage change in the number of school breakfasts and lunches served was calculated for each month and by school district need level and urbanicity level. Results: The number of school meals served decreased during the first spring of the pandemic compared to the spring of the previous school year (−43% in April, −51% in May), while the number of school meals served increased during the first summer of the pandemic compared to the summer of the previous school year (+92% in July, +288% in August). Conclusions: Waivers may provide flexibility to increase participation in school meals, especially during the summer.

## 1. Introduction

According to a report published in September 2021 by the Economic Research Service (ERS) of the United States Department of Agriculture (USDA), 10.5% of New York State (NYS) households were food-insecure across 2018–2020. This prevalence rate is similar to the national prevalence rate, which is 10.7% [1]. A report published by the New York State Health Foundation (NYSHealth) stated that, with the outbreak of the COVID-19 pandemic in the United States (US), the prevalence of food insecurity increased to 12.4% of New York households in July 2020, and food insecurity was more common in New York households with children compared to New York households without children [2]. Educational settings are critical places where children from food-insecure households access food: from April 2020 through July 2020, the most-used free food access point in NYS was schools [2]. This is despite the closure of schools in mid-March to contain the spread of the virus.

In 2020, the USDA’s child nutrition programs accounted for $21.7 billion in federal spending [3]. The child nutrition programs include the National School Lunch Program (NSLP), the School Breakfast Program (SBP), the Child and Adult Care Food Program (CACFP), and the Summer Food Service Program (SFSP). These programs, which are administered by the Food and Nutrition Service (FNS) of the USDA, provide meals and snacks to children to improve food security and dietary quality [3]. Eligibility for paid, reduced-price, or free meals and snacks is determined by the child’s household income [4]. Participation in child nutrition programs has been associated with increased food security, dietary quality, and academic performance [5]. Since the Healthy, Hunger-Free Kids Act (HHFKA) of 2010, the diet quality of school meals has improved significantly [6]. This has translated into higher quality diets for participants in school meals compared to non-participants [7,8]. According to a recent systematic review, when school meals are made free for all students, there is a positive association with diet quality [9].

The COVID-19 pandemic created barriers to participation in child nutrition programs; one key barrier was that many students were not in schools where these meals were typically served. To help overcome the barriers, FNS issued several nationwide waivers for the programs. The Meal Times Waiver allowed schools to no longer require that meals be served at regular mealtimes. The Non-congregate Feeding Waiver allowed schools to no longer require that meals be served in a group setting. The Parent/Guardian Meal Pick-Up Waiver allowed schools to no longer require that the child or children be present with the parent or guardian to pick up school meals; and the Seamless Summer Option (SSO) and Summer Food Service Operations Extension allowed schools to continue to operate SSO and SFSP during the 2020–2021 school year [10]. The advantage of SSO and SFSP over the school meal programs operated during the school year is that SSO and SFSP do not require meal payment collection. Eliminating meal payment collection enabled quicker service, reducing contact between individuals picking up school meals [11].

Despite these waivers, national data show that 1.7 billion fewer school meals were served from March 2020 to November 2020 compared to March 2019 to November 2019 [12]. Data from New York City (NYC) show that participation in school meals decreased by 68% in April 2020 [13]. National and New York City data suggest that many students across NYS may have been missing out on school meals, which are a critical source of nutrition. The objectives of this study are (1) to compare the number of school meals served by public schools in NYS during the first spring and summer of the COVID-19 pandemic to the number served before the COVID-19 pandemic, and (2) to determine relationships between the number of school meals served and the levels of school food authority (SFA) need and urbanicity.

## 2. Materials and Methods

A secondary analysis of quantitative data was conducted to compare the number of school breakfasts and school lunches served during selected months of the COVID-19 pandemic to the number served in the same months before the COVID-19 pandemic. This study was exempted from review by the Teachers College, Columbia University Institutional Review Board (New York, NY, USA).

In NYS, data from the 2018–2019 school year show that 1,434,192 students, or 55% of the students enrolled in traditional public school districts and charter schools, were eligible for a free or reduced-price lunch [14]. This study focuses on SFAs serving public schools in NYS that are also in the 2018–2019 Enrollment Database to allow comparison by need level [15]. During the 2018–2019 and the 2019–2020 school years, there were 601 unique SFAs for public schools in NYS. SFAs vary in size; a single SFA could serve meals at one site or across a district, for example.

The NYSED provided population-level data on the number of school breakfasts and lunches served per month by each SFA over the following school years, including during the summers: the 2018–2019 school year and the 2019–2020 school year. To compare the number of meals served in spring 2020 (during the COVID-19 pandemic) to the number served in spring 2019 (before the COVID-19 pandemic), we focused on data from April and May. We excluded data from March because the data we received did not indicate the number of meals served before and after the COVID-19-related school closures during March. We excluded data from June because there is wide variability in when schools hold their last day of classes in NYS, affecting the number of school days and summer days. To compare the number of meals served in summer 2020 (during the COVID-19 pandemic) to the number served in summer 2019 (before the COVID-19 pandemic), we included data from July and August. Most schools in NYS start school in September, thus all of August meals are summer meals.

The number of SFAs and proportion of SFAs serving meals were calculated by school district need level (low, average, and high) and school district urbanicity level (NYC, large cities, urban-suburban, and rural). We derived the categories for school district need level and school district urbanicity level from the NYSED’s need/resource capacity index in the 2018–2019 Enrollment Database. NYSED describes how they calculate this index in a document accompanying the database. According to NYSED, the need/resource capacity index is defined as a “measure of a district’s ability to meet the needs of its students with local resources.” The index indicates (1) the need level of a school district and (2) the urbanicity level of the community in which a school district is located. To determine need level, the “estimated poverty percentage” is divided by the “combined wealth ratio.” The estimated poverty percentage is an “approximation” of the percentage of children served by the district who qualify for free or reduced-price meals, while the “combined wealth ratio” is determined by dividing a school district’s wealth per student by the average wealth per student for the state. To determine the urbanicity level for high-need school districts, enrollment numbers and the number of students per square mile are used [15]. The analysis by urbanicity includes only schools classified as high-need (*n* = 184).

The total number of meals by month was calculated and compared from different school years (2018–2019 and 2019–2020). This same method of comparing the number of meals served by month for these school years was conducted at the national level by the US Government Accountability Office in a report published in September 2020 [16]. In our analysis, we additionally compared meals by school district need level and school district urbanicity level.

We calculated the percentage change in the number of meals served and the number of SFAs serving meals. For each term (spring and summer), we subtracted the number of school meals served across all SFAs in 2019 from the number served across all SFAs in 2020. We then divided this difference by the number served in 2019 to obtain the percentage change in the number of meals served. We repeated this analysis for each school district need level and school district urbanicity level. We also calculated the percentage change in the number of SFAs for all schools included in the analysis and by levels of school district need and school district urbanicity.

## 3. Results

In Table 1, the results are presented for all SFAs included in this analysis. In April 2019, there were 600 SFAs in NYS that provided 31,067,757 meals, and in April 2020, there were 600 SFAs that provided 17,574,921 meals. From April 2019 to April 2020, the number of SFAs did not change, but the number of meals served decreased by 43%. Similarly, in May 2019, there were 599 SFAs that provided 40,639,760 meals and in May 2020, there were 601 SFAs that provided 20,055,732 meals. From May 2019 to May 2020, the number of SFAs did not change substantially, but the number of meals served decreased by 51%. In July 2019, there were 154 SFAs in NYS that provided 6,295,475 meals, and in July 2020, there were 284 SFAs that provided 12,081,654 meals. From July 2019 to July 2020, the number of SFAs increased by 84% and the number of meals served increased by 92%. Similarly, in August 2019, there were 152 SFAs that provided 2,883,001 meals and in August 2020, there were 278 SFAs that provided 11,197,012 meals. From August 2019 to August 2020, the number of SFAs increased by 83%, and the number of meals served increased by 288%.

In Table 1, the results are also presented by school district need level. From April 2019 to April 2020, the number of SFAs remained approximately the same, while the number of school meals served decreased by 52% for SFAs in low-need districts, 21% for SFAs in average-need districts, and 50% for SFAs in high-need districts. Similarly, from May 2019 to May 2020, the number of SFAs remained approximately the same, while the number of meals served decreased for all school district need levels (−59% for SFAs in low-need districts, −36% for SFAs in average-need districts, and −55% for SFAs in high-need districts). From July 2019 to July 2020, the number of SFAs increased by 3,000% for low-need districts, 129% for average-need districts, and 30% for high-need districts. Even with this increase, only 29% of SFAs in low-need districts and only 41% of SFAs in average-need districts participated in summer meals, while 69% of SFAs in high-need districts participated in summer meals. Notably, for low-need districts, the number of SFAs increased from 1 SFA in July 2019 to 31 SFAs in July 2020. The number of school meals served also increased by 11,063% for SFAs in low-need districts, 721% for SFAs in average-need districts, and 52% for SFAs in high-need districts. Similarly, from August 2019 to August 2020, the number of SFAs increased by 3000% for low-need districts. The increase was 131% for average-need districts and 25% for high-need districts. Again, for low-need districts, the number of SFAs increased from 1 SFA in August 2019 to 31 SFAs in August 2020. The number of meals served also increased for all school district need levels (28,709% for SFAs in low-need districts, 1,515% for SFAs in average-need districts, and 207% for SFAs in high-need districts). Despite these large increases, in July 2020, SFAs were serving only a fraction of the number of meals they were serving in May 2019, which can be considered their pre-pandemic capacity. More specifically, SFAs in low, average, and high-need districts were serving only 11.2%, 29.4%, and 31.4%, respectively, of their pre-pandemic capacity.

Overall, from 2019 to 2020, there was a 25% decrease in the number of meals served by NYS public schools. This appears to be driven by decreases in the number of meals served by SFAs in low-need districts (−44%) and high-need districts (−30%).

In Table 2, the results are presented by school district urbanicity level. This analysis only includes the high-need public school districts. From April 2019 to April 2020, the number of SFAs remained the same, while the number of school meals served decreased by −64% for NYC, −48% for large cities, and −32% for high-needs urban-suburban school districts. However, the number of meals served by rural school districts increased by 9% during COVID compared to pre-COVID. From May 2019 to May 2020, the number of SFAs remained approximately the same, while the number of school meals served decreased for all school district urbanicity levels, including rural school districts (−63% for NYC, −57% for large cities, −43% for urban-suburban school districts, and −16% for rural school districts). From July 2019 to July 2020, the number of SFAs remained the same for NYC and large cities, but increased by 48% and 26% for urban-suburban school districts and rural school districts, respectively. The number of school meals served increased by 22% for NYC, 159% for large cities, 162% for urban-suburban school districts, and 246% for rural school districts. Similarly, from August 2019 to August 2020, the number of SFAs remained the same for NYC and large cities but increased by 43% and 21% for urban-suburban school districts and rural school districts, respectively. The number of school meals served also increased for all school district urbanicity levels (146% for NYC, 545% for large cities, 393% for urban-suburban school districts, and 647% for rural school districts).

Overall, among the high-need school districts, the number of meals served decreased most for NYC (−41%), followed by large cities (−19%), followed by urban-suburban districts (−18%); however, for rural high-need school districts, the number of meals served increased from 2019 to 2020 (+20%).

## 4. Discussion

Overall, the number of school meals served decreased during the first spring of the pandemic compared to the number served during the spring of the previous school year. For the analysis based on school district need, the decrease was highest for low-need and high-need school districts and schools in NYC and other large cities. Conversely, the number of school meals served increased during the first summer of the pandemic compared to the number served during the summer of the previous school year. For the analysis on school district need, the increase was highest for low-need and average-need school districts and schools in rural towns and large cities other than NYC.

When comparing April–May 2019 (pre-COVID) to April–May 2020 (during COVID), the number of school meals served in NYS decreased. This finding is similar to findings from other studies. Across three large urban school districts, including the New York City Department of Education (NYCDOE), McLoughlin et al. (2020) found a decrease in the number of school meals served per day during April 2020 when compared to usual daily meal counts. They also found that NYCDOE had the largest decrease in the number of school meals served, dropping by 32% [13]. In Connecticut, Connolly et al. (2021) reported a 28.9% decrease in the percentage of participation in school lunch during March 2020–May 2020 compared to March 2019–May 2019. Unlike our study, Connolly et al. (2021) found that the decrease from April 2019 to April 2020 was larger than the decrease from May 2019 to May 2020 [17]. In an analysis of data from ten urban school districts, Kenney et al. (2021) also found a decrease in the number of school meals served per week in the spring of 2020 relative to the prior school year [18]. In addition to these findings from the peer-reviewed literature, reports have been published on school meal participation during the pandemic. A March 2021 report from the Food Research and Action Center (FRAC) found a decrease in the number of school breakfasts and lunches served in April 2020 compared to October 2019 [19].

Qualitative research and geospatial analyses may help to explain some of our findings. In semi-structured interviews with food service directors, Connolly et al. (2021) found that communication issues and misconceptions among families may have been barriers to participation in school meals during the pandemic. For example, some families did not know about the continuation of the school meal programs and the food being served, and some did not know that they could utilize school meals along with Pandemic Electronic Benefits Transfer (P-EBT), a pandemic program that acted as an alternative to traditional school meals [17]. In interviews with members of the Urban School Food Alliance (USFA), Kenney et al. (2021) found concern that P-EBT and charitable food organizations were competing with school meals, leading to decreased school meal participation. Kenney et al. (2021) also reported neighborhood safety and parental work schedules as barriers to participation [18]. McLoughlin et al. (2020) emphasized that the decreased participation in school meals may have been related to the spread of COVID-19 at that particular time, pre-existing structural barriers, and reliance on public transportation [13]. From focus groups with parents in California, Jowell et al. (2021) reported the following barriers to participation in school meals during the pandemic: lack of access to transportation, long travel distances to meal sites, concerns about the nutritional quality of school meals, and poor communication about school meals, including lack of communication in Spanish [20]. In a two-step floating catchment area analysis of school meals sites in St. Louis, Missouri, Jabbari et al. (2021) found a decrease in site accessibility during spring 2020 compared to spring 2019 [21]. Altogether, the research suggests that there were several barriers to participation in school meals during the first spring of the pandemic.

When we compared summer, July–August 2019 (pre-COVID) to July–August 2020 (during COVID), we found that the number of school meals served in NYS increased. To our knowledge, there are no peer-reviewed studies that have analyzed the number of school meals served during the first summer of the pandemic. Instead, there are governmental and state reports on summer meals. A 2021 report from Hunger Free New Jersey found a three-fold increase in the number of school meals served in New Jersey when comparing July 2019 to July 2020 [22]. Similarly, a 2021 report from the USDA found a two-fold increase in participation in school meals in July 2020 compared to July 2019 [23].

Reports and geospatial analyses may help to explain our findings. From a survey by the University of Connecticut, 63.1% of parents reported being new to summer meals in 2020. The parents reported several reasons for participation. Among the top reasons were free meals, convenience, and positive experiences with summer meals served during the school year under the SSO and Summer Food Service Operations Extension from the USDA. The survey results suggest that other waivers may have also encouraged increased participation. For example, more than 70% of parents reported that the following flexibilities were “very important:” grab-and-go meals, the ability to pick up multiple meals at once, and the ability to pick up meals without bringing their children to the meal site [24]. Furthermore, according to a report from FoodCorps, free meals for all during the pandemic may have decreased stigma around utilization of school meal programs [25]. Prior to the Child Nutrition Area Eligibility waiver from the USDA, there may have been more stigma around utilization of the Summer Food Service Program, which required site placement in low-income communities only. Furthermore, prior to the pandemic, the Summer Food Service Program required congregate feeding, which was often described as a barrier to participation [26]. During the pandemic, the non-congregate feeding waiver may partly explain the increased participation in summer meals. Increased geographic accessibility may also partly explain increased participation: unlike in the spring, Jabbari et al. (2021) found an increase in site accessibility during summer 2020 compared to summer 2019 [21]. Beyond the waivers, the pandemic itself may be partly responsible for increased utilization of summer meals, since there was an increase in food insecurity [27] and concerns about COVID-19 exposure at grocery stores [24].

When we compared school districts by need level, we had several findings. In the spring, all school district need levels had a decrease in the number of meals served during the pandemic compared to the previous school year; however, this decrease was highest for low-need districts and high-need districts. Without more data and research, it is unclear as to why this is the case. Potential hypotheses include the following: (1) there are fewer students who qualify for free and reduced meals in low-need districts, and as a result, these students did not have a strong enough need to participate in school meals during the pandemic, (2) high-need districts may have been located in underserved communities that were resultantly more impacted by the pandemic, and as a result, there was a fear of contracting COVID at school meal sites, and (3) high-need districts may have been located in underserved communities that resultantly received other forms of food assistance, and, as a result, these other forms of food assistance competed with school meals, leading to a decrease in the number of meals served. Notably, fear of contracting COVID and competition between food assistance programs have been described in the peer-reviewed literature as factors that may be associated with decreased participation in school meals [18,20]. Moreover, since we did not have data on the number of meals served to students who qualify for free and reduced-price meals during the pandemic, we could not determine if the drop in the number of meals served in low-need districts was driven by a drop in participation by paid students. Interestingly, in the summer, the number of school meals increased across all school district need levels, but they increased the most for low-need districts and increased the least for high-need districts. Potential hypotheses include the following: (1) low-need districts did not participate in summer meals at all prior to the pandemic, but may have been required to participate during the pandemic due to executive orders issued for NYS [28,29,30,31], and (2) high-need districts may have relatively higher proportions of students participating in summer meals prior to the pandemic. Notably, our data do show a substantial increase in the number of low-need SFAs during the pandemic compared to before the pandemic. However, our data also show that, despite the executive orders [28,29,30,31], only a proportion of SFAs served meals during the summer. This was highest for the high-need districts (69% of SFAs), lower for average-need districts (41% of SFAs), and the lowest for the low-need districts (29% of SFAs). Thus, this shows that the SFAs with students that most likely were more food insecure were more likely to be serving meals.

Similarly, when we compared school districts by urbanicity level, we had several findings. In the spring, all school district urbanicity levels had a decrease in the number of meals served, except rural schools in April. Even when rural schools had a decrease in the number of meals served in May, this decrease was less than that observed for urban-suburban schools, large cities, and NYC. The decrease was highest for NYC. Potential hypotheses include the following: (1) there was less fear of the spread of COVID-19 in more sparsely populated communities, leading to increased access of school meals, and (2) NYC was the epicenter of the pandemic, and as a result, there was a fear of accessing school meal sites to obtain school meals. In the summer, the number of school meals increased across all school district urbanicity levels. The increase was highest for rural schools and lowest for NYC. The hypotheses for the spring may be applicable here as well. Additionally, summer meals are generally more difficult to access in rural areas due to limited public transportation options. Anecdotally, rural schools were delivering meals to students’ homes in NYS, and this strategy may be behind the large increases in the number of school meals served in rural areas. However, more research is needed to explore these hypotheses.

In general, our findings may have important policy implications. While we did not take measures to reduce confounding, our data suggest that the waivers may have been particularly beneficial for summer meal participation, since we observed consistent increases in the number of meals served during the first summer of the pandemic compared to the summer of the previous school year. However, the increase may be due to the combination of the waivers and the executive orders [28,29,30,31] in NYS for SFAs to serve meals during the summer. Additionally, our data suggest a continued need for school meals despite the introduction of P-EBT and the potential introduction of summer EBT. School meals confer advantages over other forms of food assistance, including the provision of a balanced, healthy meal with no requirement for cooking skills or equipment. However, some of the hypotheses generated by our study suggest that other forms of food assistance may still compete with school meals. Furthermore, some of our hypotheses suggest disparities in school meal access based on school district need level and school district urbanicity level. Altogether, these hypotheses highlight the need to consider policies that improve access to school meals. Our differential findings in the spring and summer highlight the need to consider different policies for during the school year and during out-of-school times such as the summer. During the school year, students may be described as a captive audience for school meals. Since it is difficult to achieve this same captive audience during out-of-school times, our summer findings may be considered to provide a more appropriate evaluation of the utility of the waivers for the provision of school meals in future school closures, whether these closures are routine summer closures or closures due to a public emergency or natural disaster. Overall, our study shows a decrease in the number of meals served during the spring and an increase in the summer, suggesting that the waivers issued during the pandemic may be an innovation that can improve participation in school meals during out-of-school times.

### 4.1. Strengths

Our study has several strengths. First, we used reimbursement data to analyze the change in the number of school meals served across two school years. Second, the two school years we compared allowed us to capture the influence of the pandemic and the resultant waivers. Third, we compared meal counts for two terms, the spring and summer. Fourth, our study may be the only study in the literature to compare the number of school meals served across different school district need levels and school district urbanicity levels. Fifth, our study used population-level data. Sixth, our study is on NYS, which has a prevalence of food insecurity similar to the national average [1].

### 4.2. Limitations

Our study also has several limitations. First, we did not have data on the days of service for many months and SFAs during the pandemic. This prevented us from calculating the percentage of participation. Second, we did not have data on the meal service model. We learned from our state agency that seven-day meal boxes were used by some SFAs. This missing data prevented us from relating the number of meals served to the meal service model. Future studies should consider collecting this type of data. Third, we did not capture the utilization of other food assistance programs, such as food pantries and P-EBT. Fourth, we did not have data on the number of meals served to students who qualify for free and reduced-price meals during the pandemic, so we used total meals for the pandemic and the previous school year. This did not allow us to evaluate the percentage change for students who qualify for free and reduced-price meals. Fifth, due to the study design, we cannot attribute the decreased meals counts in the spring and the increased meal counts in the summer to any specific waiver or the pandemic. Sixth, we did not have menu data and consumption data; as a result, we do not know how many students actually consumed the school meals, and we do not know if the menus were nutritious with varied options, culturally responsive, and appealing.

## 5. Conclusions

The increase in the number of school meals served in the summer and the decrease in the spring suggest that the waivers may have been particularly beneficial for summer meals. Since participation in school meals is substantially lower when school is out-of-session during the summer [32], policymakers should consider maintaining the waivers for summer meal service. This can help improve food security and prevent obesity; the rates of both increase during the summer months [33,34]. Pre-pandemic research suggests that the waivers that allowed grab-and-go meals [35] and meal time flexibility [36,37] may be particularly important for increasing participation in summer meals. Waivers may provide flexibility to increase participation in school meals, especially during the summer.

## Figures and Tables

**Table 1:** Total meal count (SFA Count) and by need level.

Month	Year	Total	Low	Average	High
April	2019	31,067,757	1,837,244	7,009,465	22,221,048
		(600)	(105)	(311)	(184)
	2020	17,574,921	882,551	5,554,809	11,137,561
		(600)	(106)	(310)	(184)
	∆	−43%	−52%	−21%	−50%
		(0%)	(1%)	(0%)	(0%)
May	2019	40,639,760	2,459,610	9,240,455	28,939,695
		(599)	(105)	(311)	(183)
	2020	20,055,732	1,013,753	5,892,778	13,149,201
		(601)	(106)	(311)	(184)
	∆	−51%	−59%	−36%	−55%
		(0%)	(1%)	(0%)	(1%)
July	2019	6,295,475	2,460	331,374	5,961,641
		(154)	(1)	(55)	(98)
	2020	12,081,654	274,607	2,720,036	9,087,011
		(284)	(31)	(126)	(127)
	∆	92%	11,063%	721%	52%
		(84%)	(3,000%)	(129%)	(30%)
August	2019	2,883,001	872	159,470	2,722,659
		(152)	(1)	(55)	(96)
	2020	11,197,012	251,215	2,576,038	8,369,759
		(278)	(31)	(127)	(120)
	∆	288%	28,709%	1,515%	207%
		(83%)	(3,000%)	(131%)	(25%)
Totals	2019	80,885,993	4,300,186	16,740,764	59,845,043
	2020	60,909,319	2,422,126	16,743,661	41,743,532
	∆	−25%	−44%	0%	−30%

**Table 2:** Total meal count (SFA Count) by high-need district urbanicity.

Month	Year	NYC	Large Cities	Urban-Suburban	Rural
April	2019	14,357,718	2,119,193	3,455,412	2,288,725
		(1)	(4)	(40)	(139)
	2020	5,201,316	1,103,011	2,335,395	2,497,839
		(1)	(4)	(40)	(139)
	∆	−64%	−48%	−32%	9%
		(0%)	(0%)	(0%)	(0%)
May	2019	18,795,832	2,767,515	4,357,244	3,019,104
		(1)	(4)	(40)	(138)
	2020	6,934,066	1,193,507	2,489,343	2,532,285
		(1)	(4)	(40)	(139)
	∆	−63%	−57%	−43%	−16%
		(0%)	(0%)	(0%)	(1%)
July	2019	4,826,574	404,657	438,521	291,889
		(1)	(4)	(21)	(72)
	2020	5,879,250	1,048,796	1,149,869	1,009,096
		(1)	(4)	(31)	(91)
	∆	22%	159%	162%	246%
		(0%)	(0%)	(48%)	(26%)
August	2019	2,259,709	164,962	188,850	109,138
		(1)	(4)	(21)	(70)
	2020	5,558,376	1,064,592	931,057	815,734
		(1)	(4)	(30)	(85)
	∆	146%	545%	393%	647%
		(0%)	(0%)	(43%)	(21%)
Totals	2019	40,239,833	5,456,327	8,440,027	5,708,856
	2020	23,573,008	4,409,906	6,905,664	6,854,954
	∆	−41%	−19%	−18%	20%

## References

[1] Coleman-Jensen A., Rabbitt M.P., Gregory C.A., Singh A. (2021). Household Food Security in the United States in 2020, Economic Research Service (ERS) of the United States Department of Agriculture (USDA).

[2] NY State of Health (2020). NYSHealth Testimony on the Impact of COVID-19 on Food Insecurity in New York State.

[3] Economic Research Service (ERS) (2020). Child Nutrition Programs, Economic Research Service (ERS) of the United States Department of Agriculture (USDA).

[4] Economic Research Service (ERS) (2022). National School Lunch Program, Economic Research Service (ERS) of the United States Department of Agriculture (USDA).

[5] Ralston K., Treen K., Coleman-Jensen A., Guthrie J. (2017). Children’s Food Security and USDA Child Nutrition Programs.

[6] Gearan E.C., Fox M.K. (2020). Updated Nutrition Standards Have Significantly Improved the Nutritional Quality of School Lunches and Breakfasts. J. Acad. Nutr. Diet..

[7] Gearan E.C., Monzella K., Jennings L., Fox M.K. (2020). Differences in Diet Quality between School Lunch Participants and Nonparticipants in the United States by Income and Race. Nutrients.

[8] Kinderknecht K., Harris C., Jones-Smith J. (2020). Association of the Healthy, Hunger-Free Kids Act With Dietary Quality Among Children in the US National School Lunch Program. JAMA.

[9] Cohen J.F.W., Hecht A.A., McLoughlin G.M., Turner L., Schwartz M.B. (2021). Universal School Meals and Associations with Student Participation, Attendance, Academic Performance, Diet Quality, Food Security, and Body Mass Index: A Systematic Review. Nutrients.

[10] Food and Nutrition Service (FNS) (2020). Child Nutrition COVID-19 Waivers, Food and Nutrition Service (FNS) of the United States Department of Agriculture (USDA).

[11] Food and Nutrition Service (FNS) (2020). CN-Extending SFSP and SSO, Food and Nutrition Service (FNS) of the United States Department of Agriculture (USDA).

[12] School Nutrition Association (SNA) (2021). New USDA Data: Fewer Meals Served, $2B Loss for School Meal Programs [Press Release].

[13] McLoughlin G.M., McCarthy J.A., McGuirt J.T., Singleton C.R., Dunn C.G., Gadhoke P. (2020). Addressing Food Insecurity through a Health Equity Lens: A Case Study of Large Urban School Districts during the COVID-19 Pandemic. J. Urban Health.

[14] New York State Education Department (NYSED) (2020). 2018–2019 Student and Educator Database. [Data Set].

[15] New York State Education Department (NYSED) (2020). 2018–2019 Enrollment Database. [Data Set].

[16] Government Accountability Office (GAO) (2020). COVID-19: Federal Efforts Could Be Strengthened by Timely and Concerted Actions.

[17] Connolly K., Babbin M., McKee S., McGinn K., Cohen J., Chafouleas S., Schwartz M. (2021). Dedication, innovation, and collaboration: A mixed-methods analysis of school meals in Connecticut during COVID-19. J. Agric. Food Syst. Commun. Dev..

[18] Kenney E.L., Dunn C.G., Mozaffarian R.S., Dai J., Wilson K., West J., Shen Y., Fleischhacker S., Bleich S.N. (2021). Feeding Children and Maintaining Food Service Operations during COVID-19: A Mixed Methods Investigation of Implementation and Financial Challenges. Nutrients.

[19] Food Research & Action Center (FRAC) (2021). School Meals: The Impact of the Pandemic on 54 Large School Districts.

[20] Jowell A.H., Bruce J.S., Escobar G.V., Ordonez V.M., Hecht C.A., Patel A.I. (2021). Mitigating childhood food insecurity during COVID-19: A qualitative study of how school districts in California’s San Joaquin Valley responded to growing needs. Public Health Nutr..

[21] Jabbari J., Chun Y., Nandan P., McDermott L., Frank T., Moreland-Russell S., Ferris D., Roll S. (2021). How Did School Meal Access Change during the COVID-19 Pandemic?. A Two-Step Floating Catchment Area Analysis of a Large Metropolitan Area. Int. J. Environ. Res. Public Health.

[22] Parello N. (2021). Hunger and Free New Jersey, New Jersey Summer Meals for Kids: 2020 Snapshot.

[23] Toossi S. (2021). COVID-19 Working Paper: Filling the Pandemic Meal Gap: Disruptions to Child Nutrition Programs and Expansion of Free Meal Sites in the Early Months of the Pandemic.

[24] UConn (2021). Feeding Connecticut’s Children during the Summer: An Evaluation of Access and Family Perspectives on Meal Sites. https://media.ruddcenter.uconn.edu/PDFs/State-wide%20summer%20meals%20report%20%283%29.pdf.

[25] Flores L., de Blasis T., Shioiri-Clark M., Polonsky H. (2020). Nourishing Learners: A Report on School Meals and Education During COVID-19.

[26] Kinsey E.W., Hecht A.A., Dunn C.G., Levi R., Read M.A., Smith C., Niesen P., Seligman H.K., Hager E.R. (2020). School Closures During COVID-19: Opportunities for Innovation in Meal Service. Am. J. Public Health.

[27] Sharma S.V., Chuang R.-J., Rushing M., Naylor B., Ranjit N., Pomeroy M., Markham C. (2020). Social Determinants of Health-Related Needs During COVID-19 Among Low-Income Households With Children. Prev. Chronic. Dis..

[28] State of New York Executive Chamber (2020). Execuztive Order 202.11 Continuing Temporary Suspension and Modification of Laws Relating to the Disaster Emergency.

[29] State of New York Executive Chamber (2020). Executive Order 202.14 Continuing Temporary Suspension and Modification of Laws Relating to the Disaster Emergency.

[30] State of New York Executive Chamber (2020). Executive Order 202.28 Continuing Temporary Suspension and Modification of Laws Relating to the Disaster Emergency.

[31] State of New York Executive Chamber (2020). Executive Order 202.4 Continuing Temporary Suspension and Modification of Laws Relating to the Disaster Emergency.

[32] Turner L., Calvert H.G. (2019). The Academic, Behavioral, and Health Influence of Summer Child Nutrition Programs: A Narrative Review and Proposed Research and Policy Agenda. J. Acad. Nutr. Diet..

[33] Huang J., Barnidge E., Kim Y. (2015). Children Receiving Free or Reduced-Price School Lunch Have Higher Food Insufficiency Rates in Summer. J. Nutr..

[34] Rundle A.G., Park Y., Herbstman J.B., Kinsey E.W., Wang Y.C. (2020). COVID-19-Related School Closings and Risk of Weight Gain Among Children. Obesity.

[35] Sather S., Schumacher J., Lanier J., Fehrenbacher J., Bardwell A. (2021). Impact of summer mobile feeding sites on increasing children’s access to food. J. Child Nutr. Manag..

[36] Chiappone A., Garvin T.M., Blaser C., Fricke H.E., Weissenburger-Moser Boyd L., Barnard T., Yaroch A.L. (2018). Factors Associated with Participation in the Oregon Summer Food Program: A Mixed Methods Analysis. J. Health Disparities Res. Pract..

[37] Harper K., Lu S.V., Gross J., Obudulu C., Wilson M.J., Gross S.M. (2022). The Impact of Waivers on Summer Meal Participation in Maryland. J. Sch. Health.

